# Overexpression of KDM5B/JARID1B is associated with poor prognosis in hepatocellular carcinoma

**DOI:** 10.18632/oncotarget.26144

**Published:** 2018-09-28

**Authors:** Yoshinobu Shigekawa, Shinya Hayami, Masaki Ueno, Atsushi Miyamoto, Norihiko Suzaki, Manabu Kawai, Seiko Hirono, Ken-ichi Okada, Ryuji Hamamoto, Hiroki Yamaue

**Affiliations:** ^1^ Second Department of Surgery, School of Medicine, Wakayama Medical University, Wakayama, Japan; ^2^ Division of Molecular Modification and Cancer Biology, National Cancer Center Research Institute, Tokyo, Japan

**Keywords:** hepatocellular carcinoma (HCC), epigenetics, histone demethylase, KDM5B/JARID1B

## Abstract

**Background & aims:**

Hepatocellular carcinoma (HCC) has high potential for recurrence, even in curative operative cases. Although several molecular-targeting drugs have been applied to recurrent HCC, their effectiveness has been limited. This study therefore aims to develop novel cancer drugs through protein methylation.

**Methods:**

We investigated the role of KDM5B/JARID1B, a member of JmjC histone demethylase, in HCC. Expression profiles of KDM5B were examined by immunohistochemical analysis in 105 HCC clinical tissue samples. To examine functional effects of KDM5B using HCC cell lines, we performed loss-of-function analysis treated with KDM5B-specific small interfering RNAs (siKDM5B).

**Results:**

All HCC cases were divided into KDM5B-positive expression group (n=54) and negative expression group (n=51). In five-year overall survival, KDM5B-positive group had poorer prognosis than KDM5B-negative (61% vs 77%, *p*=0.047). KDM5B-positive group had much poorer prognosis than that of the negative group, especially in HCC derived from persistent infection of hepatitis B virus (HBV) or hepatitis C virus (HCV) (54% vs 78%, *p*=0.015). Multivariate analysis indicated that KDM5B was the strongest risk factor for poor prognosis, especially in HCC derived from HBV/HCV. Inhibition of KDM5B could significantly suppress HCC cell proliferation through no promotion from G1 to S phase. Real-time PCR and Western blotting demonstrated that E2F1/E2F2 were downstream genes of KDM5B.

**Conclusions:**

Overexpression of KDM5B results in poor prognosis in HCC that especially derived from HBV/HCV. KDM5B appears to be an ideal target for the development of anti-cancer drugs.

## INTRODUCTION

Hepatocellular carcinoma (HCC) has high recurrence potential, even in curative operative cases. To prolong survival for advanced and/or recurrent HCC patients, sorafenib has been applied to first line chemotherapy for HCC, based on the results of the SHARP trial [1] and the Asia-Pacific trial [2]. Subsequently, a number of trials using anti-cancer drugs for HCC were completed without success [3–5]. Nonetheless, the efficacy of regorafenib for HCC patients was eventually demonstrated by the RESORCE trial [6]. Lenvatinib, a multi-kinase inhibitor, could be admitted as the first line treatment of unresectable advanced HCC in a randomized phase III non-inferiority trial [7]. Molecular-targeting drugs for HCC treatment are promising, however their effectiveness might be restricted because of the complex multi-step processes and heterogeneity of HCC [8]. It is therefore necessary to develop anti-cancer drugs for HCC patients through novel pathways.

Histone methylation is one of the most important post-translational modifications (PTMs) with phosphorylation, acetylation, sumoylation and ubiquitylation. It plays a key role in transcriptional regulation by chromatin dynamics, such as euchromatin (open chromatin) or heterochromatin (closed chromatin). In early methylation research, histone methylation was thought to be a static epigenetic modification. Identification of LSD1 (lysine-specific demethylase 1)/KDM1A in 2004, which is a histone demethylase, revealed that protein methylation is dynamically regulated [9]. A highly conserved part containing the JmjC (Jumonji C) domain was characterized to reserve histone demethylase activity [10]. KDM5B/JARID1B is also categorized as a member of Jmjc family and has demethylase activity for histone H3 lysine 4 (H3K4) di- and tri- methylation.

In 2003, SMYD3 (SET and MYND domain containing 3), a histone methyltransferase, was shown to stimulate HCC cell proliferation through its methyltransferase activity [11–15]. We have reported that various kinds of histone methyltransferases and demethylases may also contribute to human carcinogenesis [16–27]. The expression profiles of KDM5B, also known as JARID1B/PLU-1, were up-regulated significantly in various types of cancer compared with corresponding normal tissue, especially in bladder cancer and non-small cell lung cancer (NSCLC) [27]. We also found that E2F transcription factor 1 (E2F1) and E2F transcription factor 2 (E2F2) are candidate downstream modulators regulated by KDM5B in bladder cancer and NSCLC [27]. In the current study, we examined KDM5B expression status in HCC and elucidated the critical role of KDM5B in human carcinogenesis for HCC.

## RESULTS

### Clinicopathological findings of KDM5B expression in hepatocellular carcinoma

**Figure 1:**
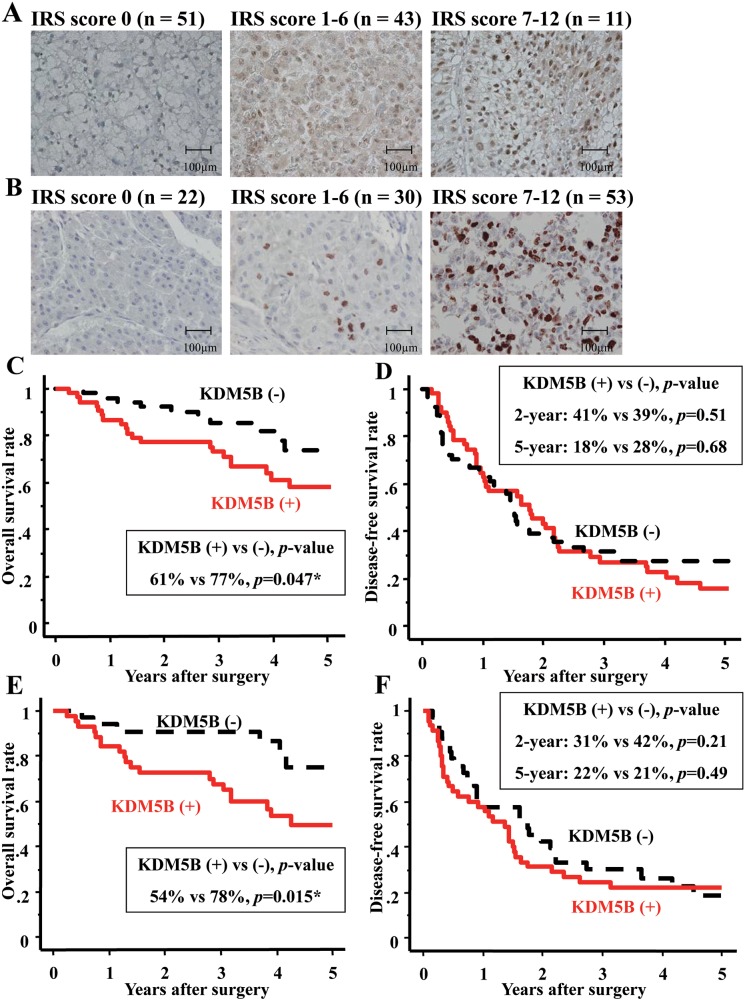
KDM5B/JARID1B was overexpressed and contributed to poor prognosis in HCC **(A)** HCC cases (n:105) were divided into three groups by immunohistochemical staining pattern of KDM5B depending on immunoreactive scoring system (IRS score) (x 400 magnification). IRS score 0 (n=51) was categorized as KDM5B-negative expression group. IRS score 1-6 (n=43) and 7-12 (n=11) were categorized as KDM5B-positive expression group (n=54). Scale bars; 100μm. **(B)** HCC cases (n:105) were divided into three groups by immunohistochemical staining pattern of Ki-67 depending on IRS score (x 400 magnification). IRS score 0 (n=22) and 1-6 (n=30) were categorized as Ki-67 negative expression group. IRS score 7-12 was categorized as Ki-67 positive expression group (n=53). Scale bars; 100μm. **(C)** KDM5B expression correlated with the five-year overall survival of HCC patients by Kaplan-Meier survival analysis (*p*=0.047). **(D)** KDM5B expression did not correlate with the two-year and five-year disease-free survival of HCC patients (*p*=0.51, 0.68, respectively). **(E)** KDM5B expression correlated with the five-year overall survival of HCC patients with hepatitis B virus antigen (HBV Ag) or hepatitis C virus antibody (HCV Ab) positive by Kaplan-Meier survival analysis (*p*=0.015). **(F)** KDM5B expression did not correlate with the two-year and five-year disease-free survival of HCC patients with HBV Ag or HCV Ab positive by Kaplan-Meier survival analysis (*p*=0.21, 0.49, respectively). ^*^p<0.05.

All HCC cases were divided into KDM5B-positive expression group (KDM5B-positive group, n=54) and KDM5B-negative expression group (KDM5B-negative group, n=51) (Figure 1A). There was no significant staining in non-neoplastic tissues and no differences between KDM5B-positive and negative groups in patient characteristics such as sex, age and alcoholic abuse (Table 1). There were more nBnC (both hepatitis B surface antigen and hepatitis C antibody are negative) patients in KDM5B-negative group than in KDM5B-positive (*p*=0.03). In preoperative laboratory findings, KDM5B-positive group had higher AFP level than KDM5B-negative group only (88.0 vs 11.5 [ng/ml], *p*=0.002) (Table 1). In pathological findings, KDM5B-positive group had poorer differentiation and more vascular invasion than KDM5B-negative group (*p*=0.04, 0.049, respectively, Table 1). A statistically significant, positive correlation was found between KDM5B and Ki-67 expressions (*p*=0.014, Figure 1B and Table 1).

**Table 1:** Clinicopathological characteristics in KDM5B positive and negative groups

Variables	all (n=105)	KDM5B positive (n=54)	KDM5B negative (n=51)	*p* value
Patient characteristics				
Sex				0.10
Male	79	37	42	
Female	26	17	9	
Age (years)	66 (33-82)	64 (38-82)	67 (33-81)	0.12
Alcoholic abuse				0.64
Yes	76	38	38	
No	29	16	13	
Hepatitis status				0.03^*^
HBV Ag positive	18	13	5	
HCV Ab positive	63	35	28	
nBnC (no infection)	27	9	18	
Preoperative laboratory data				
Albumin (g/dl)	3.9 (2.2-4.8)	3.9 (2.8-4.8)	3.9 (2.2-4.8)	0.46
Total bilirubin (mg/dl)	0.8 (0.2-2.0)	0.8 (0.2-2.0)	0.8(0.4-1.9)	0.78
Prothrombin time (%)	85.5 (51.1-113.4)	81.6 (51.1-113.4)	88.1 (52.4-109.0)	0.06
ICG R15 (%)	10.7 (1.6-36.3)	10.0 (1.6-36.3)	11.0 (1.7-36.0)	0.79
AST (IU/ml)	50 (21-166)	51 (21-166)	48 (22-147)	0.36
ALT (IU/ml)	47 (8-158)	46 (8-158)	47 (12-148)	0.84
Platelet count (x10^4^/μl)	14.5 (3.4-39.7)	14.2 (4.6-26.7)	15.5 (3.4-39.7)	0.22
Child-Pugh grade				0.54
A	92	46	46	
B	12	7	5	
C	1	1	0	
AFP (ng/ml)	34.4 (18-96575)	88.0 (1.8-96575.6)	11.5 (2.1-2978.9)	0.002^**^
AFP-L3 (%)	0.5 (0.1-89.9)	0.5 (0.1-89.9)	0.5 (0.1-85.7)	0.24
DCP (mIU/ml)	20.4 (10.0-693070)	187.0 (19.0-292000)	195.0 (10.0-693070)	0.30
Pathological characteristics				
Tumor maximum size (cm)	4.0 (1.0-15.0)	4.5 (1.0-15.0)	3.5 (1.5-11.0)	0.68
Tumor number				0.53
Single	69	37	32	
Multiple	36	17	19	
Differentiation				0.04^*^
Well	20	7	13	
Moderate	74	38	36	
Poor	11	9	2	
Vascular invasion				0.049^*^
Present	23	16	7	
Absent	82	38	44	
Fibrosis stage				0.64
4	56	30	26	
0-3	49	24	25	
Activity stage				0.97
2-3	76	39	37	
0-1	29	15	14	
TNM stage				0.62
I	9	4	5	
II	37	20	17	
III	37	15	22	
IV	22	15	7	
Ki-67 status				0.01^*^
Positive	53	37	16	
Negative	52	17	35	

^*^p<0.05, ^**^p<0.01

KDM5B: Lysine demethylase 5B; HBV: Hepatitis B virus; HCV: Hepatitis C virus; nBnC: both hepatitis B surface antigen and hepatitis C antibody are negative;ICG-R15: indocyanine green retention test at 15 minutes; AST: aspartate transaminase; ALT: alanine transaminase; AFP: alpha-fetoprotein; AFP-L3: the *lens culinaris* agglutinin-reactive fraction of AFP; DCP: des-ɤ-carboxy prothrombin.

In five-year overall survival rate after surgery, KDM5B-positive group had poorer prognosis than KDM5B-negative group (61% vs 77%, respectively, *p*=0.047, Figure 1C). There was no significant difference, however, in both two-year and five-year recurrence-free survival after surgery (KDM5B-positive vs negative, 41% vs 39%, 18% vs 28%, respectively, Figure 1D). To assess these prognostic factors, 17 variables were analyzed in the 105 patients using the Cox proportional hazards model (Table 2). Univariate analysis indicated that four variables (AFP > 20 ng/ml, vascular invasion, KDM5B-positive and Ki-67-positive) were risk factors of HCC. Consequently, in multivariate analysis, only AFP level was suggested as a prognostic factor of HCC.

**Table 2:** Univariate and multivariate analysis of prognostic factors of HCC

Variables	Univariate analysis			Multivariate analysis		
	HR	(95% CI)	*p* value	HR	(95% CI)	*p* value
Sex (male vs female)	1.84	(0.721-4.715)	0.20			
Age (≦66 vs >66)	1.08	(0.464-2.527)	0.85			
HBV infection (yes vs no)	0.76	(0.241-2.259)	0.62			
HCV infection (yes vs no)	0.44	(0.173-1.109)	0.08			
Child-Pugh grade (B or C vs A)	2.43	(0.741-7.936)	0.13			
AFP (≦20 vs >20 ng/ml)	4.33	(1.589-11.81)	0.03^*^	3.236	(1.123-9.345)	0.04^*^
AFP-L3 (≦10 vs >10%)	1.77	(0.743-4.232)	0.19			
DCP (≦40 vs >40 mIU/ml)	1.07	(0.412-2.774)	0.89			
Tumor size (≦5 vs >5cm)	1.74	(0.660-4.601)	0.26			
Triple positive (yes vs no)	1.17	(0.083-3.430)	0.68			
Tumor number (multiple vs single)	1.73	(0.722-4.149)	0.22			
Differentiation (mod/por vs wel)	2.64	(0.712-9.803)	0.14			
Vascular invasion (yes vs no)	2.52	(1.057-5.995)	0.03^*^	2.083	(1.212-5.263)	0.12
Intrahepatic metastasis (yes vs no)	2.07	(0.878-4.895)	0.09			
TNM stage (III-IV vs I-II)	1.85	(0.762-4.468)	0.17			
KDM5B (positive vs negative)	2.41	(0.995-5.843)	0.047^*^	1.738	(0.705-5.286)	0.30
Ki-67 (positive vs negative)	2.55	(1.050-6.170)	0.04^*^	1.435	(0.526-3.924)	0.58

^*^p<0.05

KDM5B: Lysine demethylase 5B; HCC: Hepatocellular carcinoma; HBV: Hepatitis B virus; HCV: Hepatitis C virus; AFP: alpha-fetoprotein; AFP-L3: the *lens culinaris* agglutinin-reactive fraction of AFP; DCP: des-ɤ-carboxy prothrombin.

There were more KDM5B-positive cases than KDM5B-negative cases in HCC cases caused by persistent infection with hepatitis B virus (HBV) or hepatitis C virus (HCV) (Table 1). These HCC cases were divided into KDM5B-positive expression (n=45) and negative expression group (n=33). There were no differences in patient characteristics (Table 3). In preoperative laboratory findings, KDM5B-positive group also had much higher AFP level than KDM5B-negative group, compared with all cases (136.2 vs 18.0 [ng/ml], *p*=0.0007) (Table 3). In pathological findings, KDM5B-positive group had larger sized tumors, poorer differentiation and more vascular invasion than KDM5B-negative group. In five-year overall survival rates after surgery, KDM5B-positive group had much poorer prognosis than KDM5B-negative group (54% vs 78%, respectively, *p*=0.015) (Figure 1E). There was no significant difference, however, in both two-year and five-year recurrence-free survival after surgery (KDM5B-positive vs negative, 31% vs 42%, 22% vs 21%, respectively, Figure 1F). Univariate analysis indicated that three variables (tumor size, vascular invasion, KDM5B-positivity) were risk factors of HCC (Table 4). In multivariate analysis, KDM5B-positivity was suggested as the strongest prognostic factor of HCC cases caused by persistent infection with HBV or HCV. For the possible reason of this poorer prognosis in KDM5B-positive group, there were more distant metastases in KDM5B-positive than in KDM5B-negative group (Table 5).

**Table 3:** Clinicopathological characteristics in KDM5B positive and negative groups for HCC derived from HBV or HCV

Variables	all (n=78)	KDM5B positive (n=45)	KDM5B negative (n=33)	*p* value
Patient characteristics				
Sex				0.08
Male	57	29	28	
Female	21	16	5	
Age (years)	66 (33-82)	64 (38-82)	67 (33-80)	0.31
Alcoholic abuse				0.38
Yes	60	33	27	
No	18	12	6	
Hepatitis status				
HBV Ag positive	18	13	5	0.15
HCV Ab positive	63	35	28	0.43
Preoperative laboratory data				
Albumin (g/dl)	4.0 (2.2-4.8)	3.9 (3.0-4.8)	4.0 (2.2-4.8)	0.65
Total bilirubin (mg/dl)	1.0 (0.2-2.0)	0.8 (0.2-2.0)	1.0 (0.4-1.9)	0.96
Prothrombin time (%)	84.0 (51.1-113.4)	81.6 (51.1-113.4)	86.1 (52.4-105.0)	0.25
ICG R15 (%)	11.0 (1.6-36.3)	11.0 (1.6-36.3)	12.0 (1.7-36.0)	0.96
AST (IU/ml)	53 (21-166)	53 (21-166)	52 (22-147)	0.60
ALT (IU/ml)	54 (8-158)	49 (8-158)	65 (20-148)	0.26
Platelet count (x10^4^/μl)	14.3 (3.4-39.7)	14.2 (5.0-26.7)	14.5 (3.4-39.7)	0.69
Child-Pugh grade				0.69
A	65	37	28	
B	12	7	5	
C	1	1	0	
AFP (ng/ml)	58.2 (18-96575)	136.2 (1.8-96575.6)	18.0 (2.1-2978.9)	0.0007^***^
AFP-L3 (%)	0.5 (0.1-89.9)	0.5 (0.1-89.9)	1.0 (0.1-85.7)	0.20
DCP (mIU/ml)	137 (10.0-693070)	187.0 (19.0-292000)	195.0 (10.0-41291)	0.05
Pathological characteristics				
Tumor maximum size (cm)	4.0 (1.0-15.0)	4.0 (1.0-15.0)	3.0 (2.0-8.0)	0.04^*^
Tumor number				0.89
Single	49	28	21	
Multiple	29	17	12	
Differentiation				0.049^*^
Well	12	5	7	
Moderate	56	31	25	
Poor	10	9	1	
Vascular invasion				0.03^*^
Present	16	13	3	
Absent	62	32	30	
Fibrosis stage				0.86
4	44	25	19	
0-3	34	20	14	
Activity stage				0.92
2-3	61	35	26	
0-1	17	10	7	
TNM stage				0.13
I	8	3	5	
II	25	15	10	
III	28	12	16	
IV	17	15	2	
Ki-67 status				0.07
Positive	43	29	14	
Negative	35	16	19	

^*^p<0.05, ^***^p<0.001

KDM5B: Lysine demethylase 5B; HCC: Hepatocellular carcinoma; HBV: Hepatitis B virus; HCV: Hepatitis C virus; ICG-R15: indocyanine green retention test at 15 minutes; AST: aspartate transaminase; ALT: alanine transaminase; AFP: alpha-fetoprotein; AFP-L3: the *lens culinaris* agglutinin-reactive fraction of AFP; DCP: des-ɤ-carboxy prothrombin.

**Table 4:** Univariate and multivariate analysis of prognostic factors for HCC derived from HBV or HCV

Variables	Univariate analysis			Multivariate analysis		
	HR	(95% CI)	*p* value	HR	(95% CI)	*p* value
Sex (male vs female)	1.77	(0.20-1.583)	0.28			
Age (≦66 vs >66)	1.17	(0.454-2.995)	0.75			
Child-Pugh grade (B or C vs A)	0.52	(0.156-1.719)	0.28			
AFP (≦20 vs >20 ng/ml)	2.85	(0.950-8.521)	0.06			
AFP-L3 (≦10 vs >10%)	1.31	(0.477-3.567)	0.61			
DCP (≦40 vs >40 mIU/ml)	1.19	(0.434-3.269)	0.73			
Tumor size (≦5 vs >5cm)	2.86	(1.064-7.692)	0.04^*^	1.824	(1.403-2.245)	0.07
Triple positive (yes vs no)	1.17	(0.083-2.430)	0.68			
Tumor number (multiple vs single)	0.57	(0.217-1.480)	0.25			
Differentiation (mod/por vs wel)	2.86	(0.609-13.40)	0.16			
Vascular invasion (yes vs no)	3.40	(1.132-10.23)	0.03^*^	1.207	(0.762-1.652)	0.23
Intrahepatic metastasis (yes vs no)	0.46	(0.177-1.184)	0.11			
TNM stage (III-IV vs I-II)	2.08	(0.776-5.586)	0.14			
KDM5B (positive vs negative)	3.60	(1.281-5.586)	0.02^*^	2.076	(1.645-2.507)	0.04^*^
Ki-67 (positive vs negative)	1.39	(0.272-1.919)	0.51			

^*^p<0.05

KDM5B: Lysine demethylase 5B; HCC: Hepatocellular carcinoma; HBV: Hepatitis B virus; HCV: Hepatitis C virus; AFP: alpha-fetoprotein; AFP-L3: the *lens culinaris* agglutinin-reactive fraction of AFP; DCP: des-ɤ-carboxy prothrombin.

**Table 5:** Recurrences pattern in both KDM5B positive and negative group

	All cases			HCC derived from HBV or HCV		
	KDM5B positive(n=54)	KDM5B negative(n=51)	*p* value	KDM5B positive(n=45)	KDM5B negative(n=33)	*p* value
Recurrence number (%)	37 (68.5)	42 (82.3)	0.20	35 (77.8)	27 (81.8)	0.66
Beyond Milan Criteria (%)	26 (48.1)	20 (39.2)	0.04^*^	19 (42.2)	12 (36.3)	0.15
Distant metastasis (%)	9 (16.7)	2 (3.9)	0.03^*^	8 (17.8)	1 (3.0)	0.01^*^

^*^p<0.05

KDM5B: Lysine demethylase 5B; HCC: Hepatocellular carcinoma; HBV: Hepatitis B virus; HCV: Hepatitis C virus.

### Growth and migration regulation of HCC cancer cells by KDM5B

**Figure 2:**
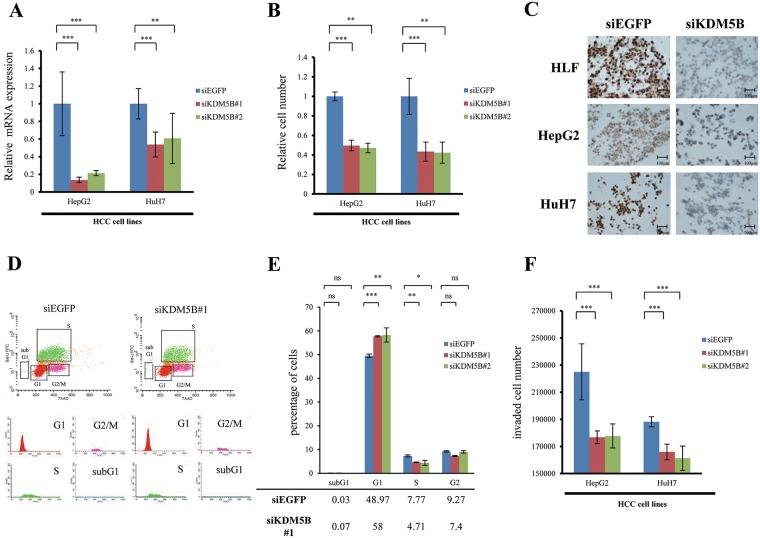
Involvement of KDM5B in the tumor growth and invasion of HCC cell lines **(A)** Quantitative real-time PCR showed the suppression of endogenous KDM5B expression of HepG2 and HuH7 using two KDM5B-specific siRNAs (#1 and #2). **(B)** KDM5B knockdown could inhibit the growth of HepG2 and HuH7 using cell growth assay. **(C)** We confirmed knockdown of KDM5B in three HCC cell lines (HLF, HepG2 and HuH7) after treatment with siKDM5B using immunohistochemical analysis. Scale bars; 100μm. **(D)** Cell cycle analysis after the treatment of siEGFP and siKDM5B. **(E)** The number of cells in each phase was counted by FACS. In KDM5B knockdown treatment, the proportion of G1 phase was larger (*p*=0.0001, 0.005 [siKDM5B#1, #2, respectively]) and that of S phase was significantly smaller than siEGFP (*p*=0.004, 0.04 [siKDM5B#1, #2, respectively]). There were no differences in the proportion of subG1 between siEGFP and siKDM5B. **(F)** Invasion assay after treatment of siEGFP and siKDM5B. All experiments were done in triplicate. Student's *t*-test was used for statistical analysis. ^*^p<0.05, ^**^p<0.01, ^***^p<0.001, ns: not significant.

For an assay of the inhibition of KDM5B, we transfected *KDM5B*-specific small interfering RNAs (siRNAs) (siKDM5B#1 and #2) and negative control (siEGFP) into two HCC cell lines whose *KDM5B* expression were highly expressed, such as HepG2 and HuH7 cells (data not shown). Expression levels of *KDM5B* in these cells transfected with siKDM5Bs were significantly suppressed compared to those transfected with siEGFP (Figure 2A). Moreover, the inhibition of KDM5B suppressed the growth of HCC cells (Figure 2B). In Figure 2C, we confirmed knockdown of KDM5B in all three HCC cell lines after treatment with siKDM5B using immunohistochemical analysis. To further assess the mechanism of this suppression, the cell cycle status of cancer cells was analyzed by flow cytometry (Figure 2D). In KDM5B knockdown treatment, the proportion of G1 phase was larger (*p*=0.0001, 0.005 [siKDM5B#1, #2, respectively]) and that of S phase was significantly smaller than siEGFP (*p*=0.004, 0.04 [siKDM5B#1, #2, respectively]) (Figure 2E). There were no differences in the proportion of subG1 between siEGFP and siKDM5B. These results indicated that KDM5B appears to progress the cell cycle from G1 to S phase and not to regulate the pathway of apoptosis. In the invasion and migration assay, the inhibition of KDM5B could suppress tumor invasion significantly (Figure 2F). These results had no conflicts with clinical data, which indicated that KDM5B-positive group had more vascular invasion than KDM5B-negative group.

### E2F1 and E2F2 worked as the downstream of KDM5B

**Figure 3:**
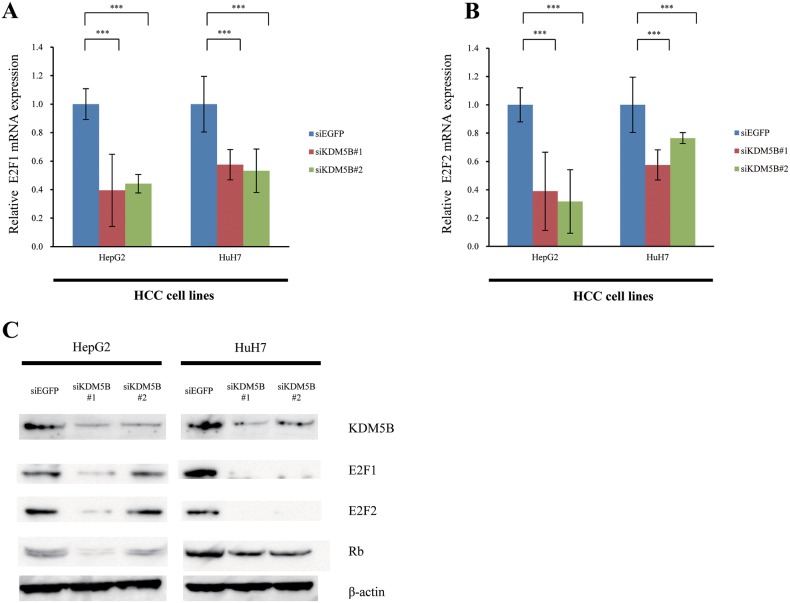
E2F1 and E2F2 are downstream genes of KDM5B **(A)** Expression levels of E2F1 in HCC cell lines (HepG2 and HuH7) were analyzed by quantitative real-time PCR after treatment with siEGFP and siKDM5B. **(B)** Expression levels of E2F2 in HCC cell lines were analyzed by real-time PCR after treatment with siEGFP and siKDM5B. **(C)** Validation of KDM5B and E2F1, E2F2 and RB expressions at the protein level. Lysates from HCC cell lines after both siEGFP and siKDM5B treatments were immunoblotted with anti-KDM5B, E2F1, E2F2, RB and actin antibodies. All experiments were done in triplicate. Student's *t*-test was used for statistical analysis. ^***^p<0.001, ns: not significant.

Referring to our previous report [27], we checked the status of *E2F1* and *E2F2* expression levels in HepG2 and HuH7 cells treated with siKDM5B (Figure 3A and 3B). Knockdown of KDM5B resulted in down-regulation of E2F1 and E2F2 mRNA expression levels. To validate the transcriptional regulation of E2F by KDM5B in more detail, we confirmed suppression of E2F1, E2F2 and RB expressions in HepG2 and HuH7 cells at the protein level (Figure 3C). These results indicated that knockdown of *KDM5B* seems to transcriptionally suppress the expression of E2F1 and E2F2, which results in suppression of cancer cell growth through inhibiting cell cycle progression.

## DISCUSSION

In this study, KDM5B-positive HCC patients showed poorer five-year overall survival rate than those in the KDM5B-negative group. KDM5B-positive group showed much poorer five-year overall survival rate than KDM5B-negative group, especially in patients with HCC caused by persistent infection of HBV or HCV. Since Lu *et al.* reported that PLU-1 was highly expressed in breast cancer [28], several reports relevant to dysregulation of KDM5B in bladder cancer [27], breast cancer [29], lung cancer [27], neuroblastoma [30], prostate cancer [31] and others [32] have been reported. In HCC, Tang *et al*. [33] and Wang *et al*. [34] reported that expression of KDM5B was up-regulated; our results correlate with these reports.

Among factors for poorer prognosis, high expression of KDM5B was the strongest prognostic factor in HCC cases caused by persistent infection of HBV or HCV. Recently, Wang *et al.* also demonstrated that KDM5B was associated with poor prognosis of HBV-related HCC and that hepatitis B virus X (HBx) protein could activate KMD5B, resulting in maintaining hepatic stem cell (HpSC)-like features in HCC [35].

The clinical characteristics of KDM5B-positive group showed elevation of AFP. We previously reported that HCC patients with triple positive tumor markers (AFP, AFP-L3 and des-ɤ-carboxy prothrombin: DCP) showed poorer prognosis due to microvascular invasion, and described the usefulness of the radiofrequency ablation (RFA) for those cases [36, 37]. Our present data show only an elevation of AFP. KDM5B-positive group also showed poorer HCC differentiation and more vascular invasion than KDM5B-negative group. These clinical data were compatible with our invasion and migration assay.

KDM5B has an enzyme activity of lysine demethylase at di-methylated and tri-methylated histone H3 lysine 4 (H3K4) [29]. On the basis of previous findings, KDM5B is likely to regulate cell proliferation and transcriptional regulation [34, 38–42]. In our results, the knockdown of KDM5B suppressed the growth of HCC cells through the block of G1-S phase transition. Moreover, there was a positive correlation between KDM5B expression and Ki-67 status. In the present study, we confirmed that E2F1 and E2F2 were down-regulated by KDM5B knockdown in HCC. E2F1 and E2F2 are members of an important group of transcription factors and we demonstrate for the first time they are also candidate downstream modulators regulated by KDM5B in HCC. Furthermore, Dai *et al.* reported that KDM5B is associated with progression of glioma by down-regulating p21, a potent cyclin-dependent kinase inhibitor (CKI) [43]. KDM5B appears to be a critical regulator of cell cycle checkpoint [34].

On the contrary, regarding disease-free survival, no statistically significant difference was observed between KDM5B-positive and KDM5B-negative patients. In our clinical data, there was no difference in the proportion of intrahepatic recurrences, even though KDM5B-positive patients showed more distant metastasis than in KDM5B-negative patients. Intriguingly, previous reports implied that KDM5B is involved in epithelial-mesenchymal transition (EMT). Kidder *et al.* reported that EMT was down-regulated during reprogramming in the absence of KDM5B [44]. Moreover, Tang *et al.* revealed that KDM5B promoted metastasis involved in the EMT via PTEN/AKT signaling [33]. In this regard, KDM5B seems to promote distant metastasis by regulating the EMT signaling pathway, which may cause poor prognosis in overall survival in the KDM5B-positive group.

One of the limitations of this study is that the number of HBV-positive cases is insufficient to conduct multivariate analysis of HBV- or HCV-positive cases independently. The number of HBV-derived HCC was small, there was no difference in HBV cases and the HBV cases with HCV cases therefore combined. Indeed, the number of enrolled HCC patients is not large, which results in the limitation of some specific statistical analysis in this study. We will accumulate more resected HCC specimens and plan further analysis.

Finally, our results clearly indicate that KDM5B is one potential molecular target for developing anti-cancer drugs. Indeed, Sayegh *et al*. developed small molecule inhibitors of KDM5B as anti-cancer agents [45], and further studies may explore the importance of KDM5B as a target of anti-cancer therapy for patients with HCC.

## MATERIALS AND METHODS

### Patients

In our study, 105 HCC patients underwent primary hepatectomy between January 2000 and December 2006 at Wakayama Medical University Hospital (WMUH). Informed consent was obtained from all patients in accordance with the guidelines of the Wakayama Medical University Ethical Committee on Human Research (approval number: 871).

### Immunohistochemistry and evaluation of their results

Pretreatment was performed as in our previous study [27]. Primary antibodies KDM5B (1:500, mouse monoclonal, clone 1G10, H00010765-M02, Abnova or HPA027179, Atlas Antibodies AB) and Ki-67 (1:1000, Rabbit-a-confirm Ki-67 MIB Clone 30-9, Ready-to-use; Ventana) were diluted in phosphate buffered saline. Antibody binding was then immuno-detected using the avidin-biotin-peroxide complex, as described by the supplier (Nichirei, Tokyo, Japan).

Immunohistochemical (IHC) staining results were evaluated by the immunoreactive scoring system (IRS score) [46–48]. The number of positive cells was evaluated numerically (0: no positive cells; 1:1-25% positive cells; 2: 26-50% positive cells; 3: 51-75% positive cells and 4: 76-100% positive cells). These scores were then multiplied with an intensity scale (0: negative; 1: weak; 2: moderate and 3: intensive staining). Valuation was performed without knowledge of the clinical-pathological variables. In accordance with previous reports, the cut-off values of IHC staining scores were set as the median value [49, 50].

### Cell culture

HepG2 and HuH7 were used as HCC cell lines that were grown in monolayers in appropriate media, Dulbecco's modified Eagle's medium (DMEM). These cells were maintained at 37°C in humid air with 5% CO_2_ condition. All experiments were done in triplicate.

### Transfection with small interfering RNAs

siRNA oligonucleotide duplexes (SIGMA Genosys, Tokyo, Japan) were prepared. These suppress expression of *KDM5B* (siKDM5B#1 and #2; sequences shown in Supplementary Table 1), as an assay of KDM5B inhibition. There was one negative control (siRNA for enhanced green fluorescent protein, siEGFP). siRNA duplexes were transfected (100 nM final concentration) to HepG2 and HuH7 with lipofectamine 2000 (Invitrogen) for 72 hours, and cells were examined for viability using Cell Counting Kit 8 (DOJINDO Laboratories).

### Quantitative real-time PCR

Specific primers for all human *g*lyceraldehyde-3-phosphate dehydrogenase (housekeeping gene), *SDH* (Succinate dehydrogenase, housekeeping gene) and *KDM5B* were designed for quantitative RT-PCR (each primer sequence in Supplementary Table 2). PCR was performed using CFX96 touch^®^ (Bio-Rad Laboratories, Hercules, CA) following the manufacturers’ protocol. Sso Advanced™ Universal SYBR^®^ Green Supermix (Bio-Rad Laboratories, Hercules, CA) (50%), 50 nM each of the forward and reverse primers and 2 μl of reversely-transcribed cDNA were applied. Similar procedures were followed to those previously reported [27].

### Cell proliferation assay

Grown on tissue culture plates, siEGFP or siKDM5B induced to HepG2 and HuH7 cells were pre-incubated in 96-well plates at a density of 15,000 cells per well with growth medium. Seventy-two hours after transfection 10 μl of Cell Counting Kit-8 solution was added. Absorbance at 450 nm was measured by microplate reader (SH-9000, Corona Electric, Ibaraki, Japan).

### Cell cycle analysis using flow cytometry assays (FACS)

HuH7 cells after treatment siEGFP and siKDM5B cultured in a CO_2_ incubator at 37°C for 72 hours. By trypsinization, 1×10^6^ cells were collected and stained with propidium iodide (PI) following the manufacturers’ instructions (Cayman Chemical Company, Ann Arbor, MI). To check the cell cycle, cells were analyzed by FACS Caliber (BD Pharmingen, San Diego, CA) with MultiCycle for Macintosh software (BD Pharmingen).

### Cell invasion assay

Transwell invasion assay was performed with reference to a previous report [51]. Briefly, 5×10^4^ cells in serum-free culture medium were added to the upper chamber of the Transwell insert (Corning Inc., Corning, NY), the lower chamber was filled with medium containing 10% Fetal bovine serum (FBS) at 37°C for 72 hours. Migrated cells through the membrane into the lower chamber were fixed by methanol and stained by crystal violet.

### Western blotting

Total protein was extracted from the cells in CelLytic M™ lysis Reagent (Merck-Sigma-Aldrich, Darmstadt, Germany). Total protein (60 μg) was transferred to nitrocellulose membrane. The membrane was proved with anti-KDM5B antibody as described in IHC, anti-E2F1 antibody (KH95, Santa Cruz Biotechnology, Dallas, TX), anti-E2F2 antibody (L-20, Santa Cruz Biotechnology) and anti-RB antibody (IF8, Santa Cruz Biotechnology). Anti-Actin (I-19, Santa Cruz Biotechnology) was used as a loading control.

### Statistical analysis

We corrected for continuity by means of Child-Pugh grade, UICC-TNM classification, or each median value of laboratory data. To measure background features, we analyzed tumor characteristics and laboratory data using Fisher's exact test and the Mann-Whitney *U* test. Survival rates were analyzed by Kaplan-Meier method and log-rank test. Risk factors for survival were analyzed using the Cox proportional hazards model. All experiments were done in triplicate and Student's *t*-test was used for statistical analysis. Statistical significance was defined as *p* <0.05.

## CONCLUSIONS

The present study clarifies that overexpression of KDM5B promotes poor survival prognosis in HCC that especially derived from HBV/HCV subgroup. Further functional analyses of KDM5B could contribute to development of novel therapeutic strategies for HCC.

## SUPPLEMENTARY MATERIALS FIGURES AND TABLES



## Abbreviations

KDM5B: Lysine (K)-specific demethylase 5B
JARID1B: Jumonji, AT-rich interacting domain 1B
HCC: hepatocellular carcinoma
HBV: hepatitis B virus
HCV: hepatitis C virus
siRNA: small interfering ribonucleic acid
E2F1/E2F2: E2F transcription factor 1/2

## References

[1] Llovet JM, Ricci S, Mazzaferro V, Hilgard P, Gane E, Blanc JF, de Oliveira AC, Santoro A, Raoul JL, Forner A, Schwartz M, Porta C, Zeuzem S (2008). Sorafenib in advanced hepatocellular carcinoma. N Engl J Med.

[2] Cheng AL, Kang YK, Chen Z, Tsao CJ, Qin S, Kim JS, Luo R, Feng J, Ye S, Yang TS, Xu J, Sun Y, Liang H (2009). Efficacy and safety of sorafenib in patients in the Asia-Pacific region with advanced hepatocellular carcinoma: a phase III randomised, double-blind, placebo-controlled trial. Lancet Oncol.

[3] Llovet JM, Decaens T, Raoul JL, Boucher E, Kudo M, Chang C, Kang YK, Assenat E, Lim HY, Boige V, Mathurin P, Fartoux L, Lin DY (2013). Brivanib in patients with advanced hepatocellular carcinoma who were intolerant to sorafenib or for whom sorafenib failed: results from the randomized phase III BRISK-PS study. J Clin Oncol.

[4] Zhu AX, Kudo M, Assenat E, Cattan S, Kang YK, Lim HY, Poon RT, Blanc JF, Vogel A, Chen CL, Dorval E, Peck-Radosavljevic M, Santoro A (2014). Effect of everolimus on survival in advanced hepatocellular carcinoma after failure of sorafenib: the EVOLVE-1 randomized clinical trial. JAMA.

[5] Kudo M, Moriguchi M, Numata K, Hidaka H, Tanaka H, Ikeda M, Kawazoe S, Ohkawa S, Sato Y, Kaneko S, Furuse J, Takeuchi M, Fang X (2017). S-1 versus placebo in patients with sorafenib-refractory advanced hepatocellular carcinoma (S-CUBE): a randomised, double-blind, multicentre, phase 3 trial. Lancet Gastroenterol Hepatol.

[6] Bruix J, Qin S, Merle P, Granito A, Huang YH, Bodoky G, Pracht M, Yokosuka O, Rosmorduc O, Breder V, Gerolami R, Masi G, Ross PJ (2017). Regorafenib for patients with hepatocellular carcinoma who progressed on sorafenib treatment (RESORCE): a randomised, double-blind, placebo-controlled, phase 3 trial. Lancet.

[7] Kudo M, Finn RS, Qin S, Han KH, Ikeda K, Piscaglia F, Baron A, Park JW, Han G, Jassem J, Blanc JF, Vogel A, Komov D (2018). Lenvatinib versus sorafenib in first-line treatment of patients with unresectable hepatocellular carcinoma: a randomised phase 3 non-inferiority trial. Lancet.

[8] Farazi PA, DePinho RA (2006). Hepatocellular carcinoma pathogenesis: from genes to environment. Nat Rev Cancer.

[9] Shi Y, Lan F, Matson C, Mulligan P, Whetstine JR, Cole PA, Casero RA, Shi Y (2004). Histone demethylation mediated by the nuclear amine oxidase homolog LSD1. Cell.

[10] Klose RJ, Kallin EM, Zhang Y (2006). JmjC-domain-containing proteins and histone demethylation. Nat Rev Genet.

[11] Hamamoto R, Furukawa Y, Morita M, Iimura Y, Silva FP, Li M, Yagyu R, Nakamura Y (2004). SMYD3 encodes a histone methyltransferase involved in the proliferation of cancer cells. Nat Cell Biol.

[12] Hamamoto R, Silva FP, Tsuge M, Nishidate T, Katagiri T, Nakamura Y, Furukawa Y (2006). Enhanced SMYD3 expression is essential for the growth of breast cancer cells. Cancer Sci.

[13] Kunizaki M, Hamamoto R, Silva FP, Yamaguchi K, Nagayasu T, Shibuya M, Nakamura Y, Furukawa Y (2007). The lysine 831 of vascular endothelial growth factor receptor 1 is a novel target of methylation by SMYD3. Cancer Res.

[14] Silva FP, Hamamoto R, Kunizaki M, Tsuge M, Nakamura Y, Furukawa Y (2008). Enhanced methyltransferase activity of SMYD3 by the cleavage of its N-terminal region in human cancer cells. Oncogene.

[15] Tsuge M, Hamamoto R, Silva FP, Ohnishi Y, Chayama K, Kamatani N, Furukawa Y, Nakamura Y (2005). A variable number of tandem repeats polymorphism in an E2F-1 binding element in the 5′ flanking region of SMYD3 is a risk factor for human cancers. Nat Genet.

[16] Cho HS, Hayami S, Toyokawa G, Maejima K, Yamane Y, Suzuki T, Dohmae N, Kogure M, Kang D, Neal DE, Ponder BA, Yamaue H, Nakamura Y (2012). RB1 methylation by SMYD2 enhances cell cycle progression through an increase of RB1 phosphorylation. Neoplasia.

[17] Cho HS, Kelly JD, Hayami S, Toyokawa G, Takawa M, Yoshimatsu M, Tsunoda T, Field HI, Neal DE, Ponder BA, Nakamura Y, Hamamoto R (2011). Enhanced expression of EHMT2 is involved in the proliferation of cancer cells through negative regulation of SIAH1. Neoplasia.

[18] Cho HS, Suzuki T, Dohmae N, Hayami S, Unoki M, Yoshimatsu M, Toyokawa G, Takawa M, Chen T, Kurash JK, Field HI, Ponder BA, Nakamura Y (2011). Demethylation of RB regulator MYPT1 by histone demethylase LSD1 promotes cell cycle progression in cancer cells. Cancer Res.

[19] Cho HS, Toyokawa G, Daigo Y, Hayami S, Masuda K, Ikawa N, Yamane Y, Maejima K, Tsunoda T, Field HI, Kelly JD, Neal DE, Ponder BA (2012). The JmjC domain-containing histone demethylase KDM3A is a positive regulator of the G1/S transition in cancer cells via transcriptional regulation of the HOXA1 gene. Int J Cancer.

[20] Hayami S, Kelly JD, Cho HS, Yoshimatsu M, Unoki M, Tsunoda T, Field HI, Neal DE, Yamaue H, Ponder BA, Nakamura Y, Hamamoto R (2011). Overexpression of LSD1 contributes to human carcinogenesis through chromatin regulation in various cancers. Int J Cancer.

[21] Kang D, Cho HS, Toyokawa G, Kogure M, Yamane Y, Iwai Y, Hayami S, Tsunoda T, Field HI, Matsuda K, Neal DE, Ponder BA, Maehara Y (2013). The histone methyltransferase Wolf-Hirschhorn syndrome candidate 1-like 1 (WHSC1L1) is involved in human carcinogenesis. Genes Chromosomes Cancer.

[22] Takawa M, Cho HS, Hayami S, Toyokawa G, Kogure M, Yamane Y, Iwai Y, Maejima K, Ueda K, Masuda A, Dohmae N, Field HI, Tsunoda T (2012). Histone lysine methyltransferase SETD8 promotes carcinogenesis by deregulating PCNA expression. Cancer Res.

[23] Toyokawa G, Cho HS, Iwai Y, Yoshimatsu M, Takawa M, Hayami S, Maejima K, Shimizu N, Tanaka H, Tsunoda T, Field HI, Kelly JD, Neal DE (2011). The histone demethylase JMJD2B plays an essential role in human carcinogenesis through positive regulation of cyclin-dependent kinase 6. Cancer Prev Res (Phila).

[24] Toyokawa G, Cho HS, Masuda K, Yamane Y, Yoshimatsu M, Hayami S, Takawa M, Iwai Y, Daigo Y, Tsuchiya E, Tsunoda T, Field HI, Kelly JD (2011). Histone lysine methyltransferase Wolf-Hirschhorn syndrome candidate 1 is involved in human carcinogenesis through regulation of the Wnt pathway. Neoplasia.

[25] Toyokawa G, Masuda K, Daigo Y, Cho HS, Yoshimatsu M, Takawa M, Hayami S, Maejima K, Chino M, Field HI, Neal DE, Tsuchiya E, Ponder BA (2011). Minichromosome maintenance protein 7 is a potential therapeutic target in human cancer and a novel prognostic marker of non-small cell lung cancer. Mol Cancer.

[26] Yoshimatsu M, Toyokawa G, Hayami S, Unoki M, Tsunoda T, Field HI, Kelly JD, Neal DE, Maehara Y, Ponder BA, Nakamura Y, Hamamoto R (2011). Dysregulation of PRMT1 and PRMT6, Type I arginine methyltransferases, is involved in various types of human cancers. Int J Cancer.

[27] Hayami S, Yoshimatsu M, Veerakumarasivam A, Unoki M, Iwai Y, Tsunoda T, Field HI, Kelly JD, Neal DE, Yamaue H, Ponder BA, Nakamura Y, Hamamoto R (2010). Overexpression of the JmjC histone demethylase KDM5B in human carcinogenesis: involvement in the proliferation of cancer cells through the E2F/RB pathway. Mol Cancer.

[28] Lu PJ, Sundquist K, Baeckstrom D, Poulsom R, Hanby A, Meier-Ewert S, Jones T, Mitchell M, Pitha-Rowe P, Freemont P, Taylor-Papadimitriou J (1999). A novel gene (PLU-1) containing highly conserved putative DNA/chromatin binding motifs is specifically up-regulated in breast cancer. J Biol Chem.

[29] Yamane K, Tateishi K, Klose RJ, Fang J, Fabrizio LA, Erdjument-Bromage H, Taylor-Papadimitriou J, Tempst P, Zhang Y (2007). PLU-1 is an H3K4 demethylase involved in transcriptional repression and breast cancer cell proliferation. Mol Cell.

[30] Kuo YT, Liu YL, Adebayo BO, Shih PH, Lee WH, Wang LS, Liao YF, Hsu WM, Yeh CT, Lin CM (2015). JARID1B Expression Plays a Critical Role in Chemoresistance and stem cell-like phenotype of neuroblastoma cells. PLoS One.

[31] Xiang Y, Zhu Z, Han G, Ye X, Xu B, Peng Z, Ma Y, Yu Y, Lin H, Chen AP, Chen CD (2007). JARID1B is a histone H3 lysine 4 demethylase up-regulated in prostate cancer. Proc Natl Acad Sci U S A.

[32] Han M, Xu W, Cheng P, Jin H, Wang X (2017). Histone demethylase lysine demethylase 5B in development and cancer. Oncotarget.

[33] Tang B, Qi G, Tang F, Yuan S, Wang Z, Liang X, Li B, Yu S, Liu J, Huang Q, Wei Y, Zhai R, Lei B (2015). JARID1B promotes metastasis and epithelial-mesenchymal transition via PTEN/AKT signaling in hepatocellular carcinoma cells. Oncotarget.

[34] Wang D, Han S, Peng R, Jiao C, Wang X, Yang X, Yang R, Li X (2016). Depletion of histone demethylase KDM5B inhibits cell proliferation of hepatocellular carcinoma by regulation of cell cycle checkpoint proteins p15 and p27. J Exp Clin Cancer Res.

[35] Wang X, Oishi N, Shimakami T, Yamashita T, Honda M, Murakami S, Kaneko S (2017). Hepatitis B virus X protein induces hepatic stem cell-like features in hepatocellular carcinoma by activating KDM5B. World J Gastroenterol.

[36] Kiriyama S, Uchiyama K, Ueno M, Ozawa S, Hayami S, Tani M, Yamaue H (2011). Triple positive tumor markers for hepatocellular carcinoma are useful predictors of poor survival. Ann Surg.

[37] Ueno M, Hayami S, Shigekawa Y, Kawai M, Hirono S, Okada K, Tamai H, Shingaki N, Mori Y, Ichinose M, Yamaue H (2015). Prognostic impact of surgery and radiofrequency ablation on single nodular HCC 5 cm: Cohort study based on serum HCC markers. J Hepatol.

[38] Tan K, Shaw AL, Madsen B, Jensen K, Taylor-Papadimitriou J, Freemont PS (2003). Human PLU-1 Has transcriptional repression properties and interacts with the developmental transcription factors BF-1 and PAX9. J Biol Chem.

[39] Madsen B, Tarsounas M, Burchell JM, Hall D, Poulsom R, Taylor-Papadimitriou J (2003). PLU-1, a transcriptional repressor and putative testis-cancer antigen, has a specific expression and localisation pattern during meiosis. Chromosoma.

[40] Benevolenskaya EV, Murray HL, Branton P, Young RA, Kaelin WG (2005). Binding of pRB to the PHD protein RBP2 promotes cellular differentiation. Mol Cell.

[41] Christensen J, Agger K, Cloos PA, Pasini D, Rose S, Sennels L, Rappsilber J, Hansen KH, Salcini AE, Helin K (2007). RBP2 belongs to a family of demethylases, specific for tri-and dimethylated lysine 4 on histone 3. Cell.

[42] Chicas A, Kapoor A, Wang X, Aksoy O, Evertts AG, Zhang MQ, Garcia BA, Bernstein E, Lowe SW (2012). H3K4 demethylation by Jarid1a and Jarid1b contributes to retinoblastoma-mediated gene silencing during cellular senescence. Proc Natl Acad Sci U S A.

[43] Dai B, Hu Z, Huang H, Zhu G, Xiao Z, Wan W, Zhang P, Jia W, Zhang L (2014). Overexpressed KDM5B is associated with the progression of glioma and promotes glioma cell growth via downregulating p21. Biochem Biophys Res Commun.

[44] Kidder BL, Hu G, Yu ZX, Liu C, Zhao K (2013). Extended self-renewal and accelerated reprogramming in the absence of Kdm5b. Mol Cell Biol.

[45] Sayegh J, Cao J, Zou MR, Morales A, Blair LP, Norcia M, Hoyer D, Tackett AJ, Merkel JS, Yan Q (2013). Identification of small molecule inhibitors of Jumonji AT-rich interactive domain 1B (JARID1B) histone demethylase by a sensitive high throughput screen. J Biol Chem.

[46] Remmele W, Stegner HE (1987). [Recommendation for uniform definition of an immunoreactive score (IRS) for immunohistochemical estrogen receptor detection (ER-ICA) in breast cancer tissue]. [Article in German]. Pathologe.

[47] Rogenhofer S, Kahl P, Mertens C, Hauser S, Hartmann W, Buttner R, Muller SC, von Ruecker A, Ellinger J (2012). Global histone H3 lysine 27 (H3K27) methylation levels and their prognostic relevance in renal cell carcinoma. BJU Int.

[48] Aigelsreiter A, Ress AL, Bettermann K, Schauer S, Koller K, Eisner F, Kiesslich T, Stojakovic T, Samonigg H, Kornprat P, Lackner C, Haybaeck J, Pichler M (2013). Low expression of the putative tumour suppressor spinophilin is associated with higher proliferative activity and poor prognosis in patients with hepatocellular carcinoma. Br J Cancer.

[49] Campbell EJ, McDuff E, Tatarov O, Tovey S, Brunton V, Cooke TG, Edwards J (2008). Phosphorylated c-Src in the nucleus is associated with improved patient outcome in ER-positive breast cancer. Br J Cancer.

[50] Cappia S, Righi L, Mirabelli D, Ceppi P, Bacillo E, Ardissone F, Molinaro L, Scagliotti GV, Papotti M (2008). Prognostic role of osteopontin expression in malignant pleural mesothelioma. Am J Clin Pathol.

[51] Albini A, Iwamoto Y, Kleinman HK, Martin GR, Aaronson SA, Kozlowski JM, McEwan RN (1987). A rapid *in vitro* assay for quantitating the invasive potential of tumor cells. Cancer Res.

