# Supplementary material for: Overexpression of KDM5B/JARID1B is associated with poor prognosis in hepatocellular carcinoma
Source: Oncotarget. 2018 Sep 28;9(76):34320–35. doi: 10.18632/oncotarget.26144 (PMC6188148; doi:10.18632/oncotarget.26144)
Supplement: Supplementary file 1 [file oncotarget-09-34320-s001.pdf]

Overexpression of KDM5B/JARID1B is associated with poor prognosis in hepatocellular carcinoma

SUPPLEMENTARY MATERIALS

Supplementary Table 1: siRNA sequences

siRNA name	Sequence
siEGFP	Sense: 5’ GCAGCACGACUUCUUCAAGTT 3’
	Antisense: 5’ CUUGAAGAAGUCGUGCUGCTT 3’
siKDM5B#1	Sense: 5’ CAGUGAAUGAGCUCCGGCATT 3’
	Antisense: 5’ UGCCGGAGCUCAUUCACUGTT 3’
siKDM5B#2	Sense: 5’ GGAAUAUGGAGCUGACAUUTT 3’
	Antisense: 5’ AAUGUCAGCUCCAUAUUCCTT 3’

Supplementary Table 2: Primer sequences for real-time PCR

Gene name	Primer sequence
GAPDH (housekeeping gene) -f	5’ GCAAATTCCATGGCACCGTC 3’
GAPDH (housekeeping gene) -r	5’ TCGCCCCACTTGATTTTGG 3’
SDH (housekeeping gene) -f	5’ TGGGAACAAGAGGGCATCTG 3’
SDH (housekeeping gene) -r	5’ CCACCACTGCATCAAATTCATG 3’
KDM5B -f	5’ ATTGCCTCAAAGGAATTTGGCAGTG 3’
KDM5B -r	5’ CATCACTGGCATGTTGTTCAAATTC 3’
E2F1 -f	5’ GCTGGACCACCTGATGAATATC 3’
E2F1 -r	5’ TCTGCAATGCTACGAAGGTCCTG 3’
E2F2 -f	5’ TGGCAACTTTAAGGAGCAGACAG 3’
E2F2 -r	5’ GGGCACAGGTAGACTTCGATGG 3’
